# Age inclusive services or separate old age and working age services? A historical analysis from the formative years of old age psychiatry *c.*1940–1989

**DOI:** 10.1192/pb.bp.113.046250

**Published:** 2015-04

**Authors:** Claire Hilton

**Affiliations:** 1 CNWL NHS Foundation Trust, Harrow

## Abstract

The Equality Act 2010 made it unlawful to discriminate in the provision of services on the grounds of age. This legislation is open to interpretation, but it is affecting the way older people’s services are defined and provided. Historical evidence indicates that, since the 1940s, apart from psychiatrists working in dedicated old age services, most were unenthusiastic about working with mentally unwell older people and unsupportive of those who chose to do so. A historical analysis might shed light on current dilemmas about ‘all age’ or ‘old age’ services and inform decision-making on future mental health services.

The Equality Act 2010^1^ made it unlawful to discriminate in the provision of services on the grounds of chronological age, but precisely how the Act can be brought into effect to produce appropriate services for older people is unclear. The Act has led to reconsideration of whether dedicated services for older people, or ‘all age’ services, are clinically most appropriate and legal, and to the Royal College of Psychiatrists Old Age Faculty proposing new criteria to define their specialty.^2^ Key themes in public health policy and practice recur and are re-addressed by each generation.^3^ Some reiteration of themes is inevitable and sensible in the context of material changes in society and new medical discoveries. But attitudes in society can be slow to change and some deep-seated cultural beliefs endure, which, although not reproduced identically at different times, provide antecedents of public resistance to health innovation. Historical analysis can help clarify this process^4^ and may facilitate rational decision-making. This is particularly important for new policies as ‘policy makers are constrained from behaving rationally in many ways’: they, like the rest of society, are not value-free and they are faced with influences of past policy that may restrict options.^5^ This paper derives from a historical study concerning the formative years of the specialty of old age psychiatry until it was recognised by the Department of Health in 1989. It would be difficult to undertake a reflective historical analysis of the years 1990–2014, partly because of its recency and also because of methodological difficulties such as accessing government documents under the ‘30 year rule’. Historical evidence from the formative years may nevertheless help inform current decision-making on planning mental health services for older people.

## The very beginning: the 1940s

There was little enthusiasm for working specifically with older mentally ill people in the 1940s: ‘After all, they had to consider the medical personnel as well as the patients’^6^ one psychiatrist commented at a psychiatry conference. Geriatric medicine was new, and contrary to cultural beliefs that emphasised inevitable decline in old age, the early geriatricians demonstrated that age-appropriate specialist treatment could reverse much physical impairment.^7^ Birth rates had been falling and infant mortality increased at the beginning of the Second World War.^8^ Population projections were that one in six of the population would be of pensionable age by 1961. Welfare planners were not to know that this was a significant overestimate. The government prioritised supporting families and young children and William Beveridge’s plan for the welfare state gave older people less priority.^9^

Joseph Sheldon’s study of older people noted their ‘mental vigour and ”guts’”, and concluded that ‘living in the environment they are used to, of having something to do, and of being still able to feel necessary to the world’ were important for their well-being.^10^ This was an innovative message for health and social care authorities, who tended to focus on the minority who needed intense support, rather than the well majority. Such evidence challenged widespread negative assumptions,^11^ including in the medical profession that tended to neglect mentally ill older people.^12^

## The first old age psychiatry services *c.*1950–*c.*1970

In the 1950s, dedicated old age services were almost nonexistent. A report in the *Guardian* commented that mental hospitals ‘should be regarded as treatment centres for the mentally ill and not as depositing grounds for the senile for whom nothing can be done’.^13^ The very presence of older people in mental hospitals was deemed, by some psychiatrists, to undermine care for those ‘more in need of active treatment, having to be denied admission’,^14^ i.e. younger people. Brice Pitt (later professor of old age psychiatry) said: ‘The hospital... was like a castle, a good registrar would fend off the elderly, as those who got in were bound to stay, bound to be dumped by their family’.^15^

Once admitted, older people mainly by-passed the assessment wards and were admitted directly to long-stay back wards.^16^ Potentially reversible physical and psychiatric disorders often remained undiagnosed.^17^ Grudging medical attention given to older people^18^ contrasted with that given to younger people on forward-looking wards that became hotbeds of intervention with new treatments^19^ and rehabilitation.^20^ Emil Kraepelin’s influential opinion prevailed: most old age psychiatric disorder was irreversible senile dementia,^21,22^ the ‘result of the natural wear and tear of the body’.^23^ This stereotypical view of the inevitability of decline undermined proactive approaches to treatment. Evidence from geriatric medicine about treatment, rehabilitation and support for families^24^ had not permeated mental hospital practice. Psychiatrists working across all ages offered little to older people.

In the early 1950s, at Crichton Royal Hospital, Dumfries, rather than admitting older people directly to back wards, in order to improve outcome, all patients regardless of age began to be admitted to acute admission wards. This all age approach failed: the admission wards filled with older people. Their needs were different: different presentations of the same illnesses, different sensitivities to medication, slower rehabilitation and negative staff expectations of recovery could undermine treatment. Also, mixing older, frail, restless and confused people with acutely disturbed younger people was not conducive to safety or well-being.^16,25^ In response, at Crichton Royal in 1958, Ronald (Sam) Robinson established a dedicated comprehensive old age psychiatry service, a successful prototype for others to emulate.^26^

In 1959, the medical committee at the Bethlem-Maudsley psychiatric teaching hospital in south London analysed recent publications about the needs of older people (see, for example, National Old People’s Welfare Coucil^27^). Several consultants were consulted in this analysis, but Felix Post, the only consultant working specifically with older people, was not.^28^ Reasons were not given, but perhaps an old age psychiatrist’s opinion was not considered relevant, implying that the really important clinicians worked with younger people. This mirrored leaving out geriatricians from a British Medical Association committee in 1954 when planning geriatric services^29^ and later events at the Royal College of Psychiatrists.^30^ The Bethlem-Maudsley committee concluded that better services for older people would relieve ‘pressure caused by aged and infirm people’ in mental hospitals: this would give more scope for treating younger patients. Post repeatedly tried to improve services for his patients during the 1950s and 1960s, an impossible challenge for a lone psychiatrist advocating for older people. The Bethlem-Maudsley’s prioritisation of younger people and ambivalence towards older people persisted. It included proposals to reduce beds in the old age wards and re-designate them to other departments,^31^ and seemed to perpetuate an inferior share of resources for older people.

Elsewhere, there was also ambivalence among general psychiatrists towards treating older people. At Claybury Hospital, Essex, in 1966, some general consultants wanted to keep their older patients with treatable conditions, but hand over those thought to be incurable: ‘the general psychiatrists were dead keen to get us to take their old schizophrenics’ recollected Pitt. Others wanted to keep their caseload of older people perceived as needing little clinical input: it conveniently boosted their numbers.

In all age services, where no old age psychiatrist effectively advocated for older people, they were treated inequitably; similar happened at policy level. For example, the Worcester Development Project, a feasibility study of comprehensive community and district general hospital mental health services to replace a local psychiatric hospital, did not automatically include older people.^32^ In Northern Ireland, in 1970, the Ministry commented that older people ‘often have to go through the general admission unit, to the distress of younger patients and the detriment of the service’.^33^ It did not mention that older people might be distressed by younger patients, or that the NHS was meant to be universal.

## Old age services begin to increase: 1970s

In the 1970s, there was a tendency to plan for younger before older mentally ill people. *Hospital Services for the Mentally Ill*, in 1971, mentioned ‘psycho-geriatric’ assessment but did ‘not deal with services for elderly patients whose mental illness symptoms are the result of ageing or physical disease or both’. It promised further guidance.^34^ The mental health charity MIND and the Royal College of Psychiatrists were disappointed by the exclusion.^35,36^ Not mentioning older people did not necessarily exclude them, but left ambiguities rather than a sense of direction and responsibility for provision. It conveyed that their particular needs were unimportant and discouraged the development of specific services, despite increasing clinical evidence of benefits from active interventions.^37,38^

A geriatrician noted that psychiatric hospitals were becoming ‘silted up’ with older mentally ill people, at least partly because psychiatrists ‘do not wish to treat’ them.^39^ Tony Whitehead, an old age psychiatrist, commented that psychiatrists should stop ‘pretending that the old were not their concern’.^40^ The logical course to avoid competing with younger people was to provide dedicated, resourced services.^41^ In 1972, the Department of Health and Social Security’s (DHSS) *Services for Mental Illness Related to Old Age*, based on recent clinical experience in a few places, recommended appointing a psychiatrist with ‘special responsibility’ for older people in each health authority catchment area.^42^ Progress was slow.^43^ The DHSS acknowledged that ‘old people are almost inevitably neglected among the competing demands of acute work with younger people’^44,45^ and, in 1976, reiterated the need for ‘at least one consultant in each district’ to lead clinically and to develop services for older people.^46^

The Royal College of Physicians of London suggested reasons for underprovision of dedicated services for geriatric medicine that were equally applicable to old age psychiatry. They included the need for adequate resources and ‘fundamental changes in society’s attitude to old people’.^47^ The general psychiatric leadership of the Royal College of Psychiatrists made disparaging comments about old age psychiatry: it might create ‘a vacuum for unsuitably qualified people’ and ‘It was necessary to preserve standards and maintain some unity’.^48^ These comments implied their view that ‘psychogeriatrics is a dead end job for which no psychiatrist in his right mind applies’.^49^ Their stereotypical attitudes revealed prejudices about working with older people.

Older people accumulated in mental hospitals. In 1978, in England and Wales, people over 75 years old occupied 20 000 mental hospital beds (25%),^50^ but that age group accounted for only 5.6% of the total population.^51^ To some degree, the need for institutional care reflected age-related degenerative disorders and the needs of ageing long-stay patients with illnesses dating back many years. Other factors affecting bed use were less justifiable: clinically unnecessary admissions; low expectations of recovery; patients remaining in hospital when they no longer required treatment and they could have been better supported elsewhere; limited provision of domiciliary services and community residential care; unsupported families being unable to cope with the care of older people;^52^ and potentially curable conditions such as depression remaining undiagnosed.^53^ In addition, they were given less opportunity than younger people for community and rehabilitation services, probably at least partly related to their perceived low economic value,^54^ a yardstick of success in Western society.

## Into the 1980s

Attitudes to illness and disability in old age did not generally improve, at least not sufficiently to influence service provision widely. A joint geriatric, psychiatric and nursing report in 1987 noted that ‘the low worth of old people and their therapeutic potential still persist despite the dramatic evidence to the contrary’.^55^ Attitudes outside old age specialties were often derisory, in contrast to the rewarding nature of the work experienced by staff doing the job.^56,57^ A lecturer at a nursing conference in 1982 commented: ‘the thought of being permanently posted to a psychogeriatric ward fills newly qualified nurses with dread’.^58^ The president of the Royal College of Psychiatrists, Thomas Bewley, commented: ‘it was quite difficult to discover what percentage of a psychiatrist’s sessions were spent on caring for old people, part of the problem being that the general psychiatrist might have fears about being labelled as a psychogeriatrician’.^59^

In some places, general psychiatrists were unwilling to share resources with those taking responsibility for older people;^60^ resources might have been even less for older people if no one was specifically advocating for them. For example, the admired old age psychiatric service at Redruth, Cornwall, which hosted numerous visiting dignitaries, including Prime Minister Edward Heath and teams from abroad, consistently faced ‘half-hearted understanding rather than fervent support’ from the local general psychiatrists.^61^ The precise level of resources was probably less important than local colleagues’ willingness to offer a commensurate share of existing resources. The problem of providing adequately for older people meant that old age psychiatrists’ roles included: ‘occasional militancy... to gain a fair share of scant resources, to put them to best use, to make do with too little while wheeling, dealing, and fighting for more’.^62^ As in earlier decades, meetings about old age psychiatry service development took place without old age psychiatrists and risked overlooking older people’s mental illnesses.^63,64^

There were few formal studies comparing clinical effectiveness of old age and all age psychiatric services. One study conducted in 1985/6 compared ‘specialised’ and ‘non-specialised’ services treating older people with mental illness. Despite difficulties in the sampling method, which were likely to minimise differences, it indicated outcomes ‘in favour of the specialised services’, such as for teaching, research and having beds in general hospitals rather than in psychiatric hospitals,^65^ suggesting that dedicated services provided more forward-thinking services. Methodologically ideal randomised studies were lacking, and even well-designed studies risked creating artificial environments of case selection, staffing levels and case-load.^66^ Comparative studies often did not state clinical outcomes,^67^ although some noted better outcomes for depression treated by old age services.^68^ Better outcomes could irritate colleagues of equal status who do not like to be told by others that they can do the job better: challenges to professional skills are linked with ambivalence towards emerging new specialties,^69^ risking undermining developments.

General practitioners (GPs) mentioned advantages of having old age psychiatric services ‘closely allied’ to primary healthcare, but this was sometimes almost synonymous with shortage of resources.^70^ Where dedicated old age mental health services existed, GP referral rates of older people increased significantly: at Crichton Royal, between 1974 and 1984, a 16% increase in the population over 65 was associated with a 150% increase in referrals,^71^ suggesting that GPs valued the interventions provided.

## Discussion

‘Age’ can be measured in different ways.^72^ Usually in health service planning, ‘chronological age’ was used. A ‘chronological’ retirement age is arbitrary, but men’s retirement and pension age, 65 years, was socially acceptable to define and establish services for older people.^73^ In clinical work, staff knew the limits of their responsibilities and it could constructively guide GPs to refer patients to the appropriate psychiatric team. Chronological age cut-offs remain inseparable from certain services. They are used at both ends of life for administrative matters such as for leaving school or receiving a state pension or to enable population needs to be estimated and plans implemented. Age-related physiological and social factors affect illness in old age and may interact, requiring a distinct body of clinical knowledge and skills to permit optimum treatment. Illnesses in childhood and adolescence also present differently from in adulthood with different diagnostic and treatment implications. Not all adolescents reach ‘maturity’ at the same chronological age, and not all older people age at the same rate. In childhood and adolescence, separate services based on chronological age are acceptable. Similarly, old age services may be necessarily and appropriately different, rather than bearing overtones of negative discrimination.

An alternative definition of age is ‘cultural age’, combining chronological age with aspects of function (‘functional age’), degree of independence and capacity for self-care, coupled with the understanding of old age within a community’s value system.^74^ It relates to society’s expectations of outcome of treatment and priorities about providing health services. Those factors are not neutral and might affect providing and planning services for older people.

How best to ensure non-discriminatory services for older people remains unclear. In line with their understanding of the Equality Act, the Royal College of Psychiatrists’ Old Age Faculty has proposed criteria for services based on ‘cultural’, rather than ‘chronological’, age.^2^ This might, however, be discriminatory, given the subjective implications of the former, and since it raises issues of whether it could reliably provide appropriate services for people who need them. Who would decide on robust clinical grounds, for example, which patients should be referred to which service? Ambiguous lines of responsibility for older patients might contribute to undermining treatment for them.

Negative attitudes towards treating older people persist widely in the NHS and there is evidence that older people are still unwelcome in hospitals.^75–77^ Times have changed, legislation has changed, but attitudes appear similar. The World Psychiatric Association recently noted that combating ageism was part of the remit of services for older people;^78^ taking that perspective might be difficult for those also advocating for younger people, especially where there are resource constraints. This reflects the historical evidence that services improved where dedicated old age psychiatrists advocated for their patients and were listened to.

General psychiatrists have repeatedly demonstrated a lack of interest and desire to work with older people and excessively low expectations of health improvement for them. Clinicians, managers and NHS planners overlooked their needs when creating services and allocating resources. A survey of health service commissioners in 2010 identified a disconcerting pattern of government response: ‘Governments and commissioners have shown a surprising failure to realise the significance of the ageing population, adopt best practice and make service development for older people a national priority’.^79^

Services for older people lag behind those for younger people (see, for example, Hilton^80^). Since the 1970s, policies for older people have appeared after those for younger people, reinforcing the idea that the needs of older people are less pressing. The National Service Frameworks for mental health in 1999 and for older people in 2001^81^ are more recent examples. Two years might seem little, but in the context of this being a repeated pattern, with reluctance to provide for older people, and in the context of economic downturns, including the financial crisis beginning in 2008, this has probably cumulatively undermined service development. A recent government strategy for mental health stated: ‘we will use the word ”people” to encompass infants, children, young people, working-age adults and older people’.^82^ Age-equality is welcome, but has the anti-discrimination agenda become so all-encompassing and the policies so inclusive and watered down as to be meaningless? Will this pattern prevent real differences from being recognised? All age government strategies, however, have the potential to enhance equality in terms of planning services and allocating resources to all age groups simultaneously. By contrast, evidence of negativity towards older people by general and all age psychiatrists reinforces the importance and appropriateness of clearly defined, chronologically age-based, separate services to ensure reliable, dynamic, enthusiastic and effective psychiatric provision in old age.
